# Treating Negative Symptoms in Schizophrenia: an Update

**DOI:** 10.1007/s40501-016-0075-8

**Published:** 2016-04-08

**Authors:** Gary Remington, George Foussias, Gagan Fervaha, Ofer Agid, Hiroyoshi Takeuchi, Jimmy Lee, Margaret Hahn

**Affiliations:** Department of Psychiatry, University of Toronto, Toronto, ON Canada; Institute of Medical Science, University of Toronto, Toronto, ON Canada; Centre for Addiction and Mental Health (CAMH), 250 College St., Toronto, ON M5T 1R8 Canada; Department of Neuropsychiatry, Keio University School of Medicine, Tokyo, Japan; Department of General Psychiatry 1, Institute of Mental Health, 10 Buangkok View, 539747 Singapore, Singapore

**Keywords:** Schizophrenia, Negative symptoms, Treatment, Pharmacotherapy, Brain stimulation

## Abstract

Interest in the negative symptoms of schizophrenia has increased rapidly over the last several decades, paralleling a growing interest in functional, in addition to clinical, recovery, and evidence underscoring the importance negative symptoms play in the former. Efforts continue to better define and measure negative symptoms, distinguish their impact from that of other symptom domains, and establish effective treatments as well as trials to assess these. Multiple interventions have been the subject of investigation, to date, including numerous pharmacological strategies, brain stimulation, and non-somatic approaches. Level and quality of evidence vary considerably, but to this point, no specific treatment can be recommended. This is particularly problematic for individuals burdened with negative symptoms in the face of mild or absent positive symptoms. Presently, clinicians will sometimes turn to interventions that are seen as more “benign” and in line with routine clinical practice. Strategies include use of atypical antipsychotics, ensuring the lowest possible antipsychotic dose that maintains control of positive symptoms (this can involve a shift from antipsychotic polypharmacy to monotherapy), possibly an antidepressant trial (given diagnostic uncertainty and the frequent use of these drugs in schizophrenia), and non-somatic interventions (e.g., cognitive behavioral therapy, CBT). The array and diversity of strategies currently under investigation highlight the lack of evidence-based treatments and our limited understanding regarding negative symptoms underlying etiology and pathophysiology. Their onset, which can precede the first psychotic break, also means that treatments are delayed. From this perspective, identification of biomarkers and/or endophenotypes permitting earlier diagnosis and intervention may serve to improve treatment efficacy as well as outcomes.

## Introduction

Paralleling the advent of the second generation or “atypical” antipsychotics (SGAs) in the 1990s has been several important shifts in how schizophrenia is conceptualized. First, it has transitioned from an illness defined by psychosis to one reflecting multiple symptom domains. Interest in negative symptoms had been generated by work through the 1980s, but the picture broadened to include, in particular, cognitive symptoms. Second, discussions regarding outcome began to firmly embrace the notion of functional versus clinical recovery, the latter historically enmeshed in resolution of positive symptoms (e.g., hallucinations, delusions). The third shift related to antipsychotics themselves. Early evidence was seen as indicating that the newer drugs, with their different pharmacology, could effectively treat these other domains, giving rise to the notion that they were much more than anti-psychosis agents.

With over two decades of clinical experience involving these newer drugs now behind us, there have been further changes in thinking. Enthusiasm regarding the clinical benefits of SGAs for symptoms beyond psychosis has been tempered considerably based on accumulated evidence [1]. At the same time, the relevance of both negative and cognitive symptoms in terms of functional recovery [2] has become central to rapidly expanding efforts focused on: (a) better understanding these domains and (b) establishing effective treatments. Indeed, it is not uncommon to see new treatments evaluated on both domains. However, the focus here will be negative symptoms, an updated appraisal of somatic treatments over the last several years, and an overview of challenges and opportunities as we move forward. The gold standard will be meta-analyses where negative symptoms have been the primary outcome, followed by more global meta-analyses and recent randomized controlled trials (RCTs).

## Negative symptoms

Central to the work arising out of the 1980s was the distinction between primary (i.e., deficit) and secondary negative symptoms [3], although clinically they can be indistinguishable. More recently, the National Institute of Mental Health (NIMH) Measurement and Treatment Research to Improve Cognition in Schizophrenia (MATRICS) turned its attention to negative symptoms. From a treatment perspective, it is noteworthy that the MATRICS consensus statement makes reference to persistent negative symptoms and indicates the distinction between primary and secondary negative symptoms is not essential [4]. In addition, the following features are identified: affective flattening, alogia, avolition, asociality, and anhedonia [4, 5]. Factor analyses have isolated two separate but related subdomains, diminished expression (e.g., affective flattening), and amotivation (e.g., avolition/apathy) [6].

While debate continues regarding the interrelationship and contribution of negative versus cognitive (both social cognition and neurocognition) symptoms to functional impairment, it is clear that their impact is substantial. Resolution of positive symptoms, even in the early stages of schizophrenia, does not necessarily translate to functional recovery; figures suggest that full functional/social recovery occurs in less than 15 % of individuals with schizophrenia, with negative symptoms playing a significant role [7]. To this last point, work focusing on the prodrome of schizophrenia indicates that deficit symptoms and cognitive impairment are evident by the time of the first psychotic break [8, 9], while enduring primary negative symptoms have been identified in approximately 25–30 % of individuals with chronic schizophrenia [10].

## Treatment

The very nature of negative symptoms has implications regarding pathophysiology and treatment. Whereas positive symptoms are framed in the context of excess activity (e.g., hyperdopaminergia), negative symptoms, at least historically, have been conceptualized as reflecting a loss of functioning [11]. This position was central to the early work distinguishing positive and negative symptoms, where negative symptoms were associated with structural CNS changes. As a result, it was postulated that these features would not be amenable to pharmacotherapy in the same fashion as positive symptoms [12]. This said, how negative symptoms are conceptualized has shifted and numerous lines of investigation have been undertaken based on the notion that somatic as well as non-somatic interventions may well effect improvement in the so-called primary negative symptoms.

The level of interest and enthusiasm are reflected in the number of reviews on this topic in just the last 2 years, with a cluster of these, including a recent meta-analysis, specifically addressing treatment [13, 14, 15••, 16–18]. This article follows the format of at least some of these recent reviews, summarizing and updating the research according to psychiatric medication class (antipsychotics, antidepressants, CNS stimulants, anticonvulsants) or highlighted site/mechanism of action (glutamate, acetylcholine, serotonin, sex hormones, inflammation-immunology). Brain stimulation is also reviewed, although non-somatic treatments are not; the reader is referred to several of the aforementioned reviews for such information [16, 18]. Finally, it is important to highlight the fact that these different interventions are, as a rule, carried out in the face of concomitant antipsychotic treatment, the exception being trials involving newer antipsychotics.

### Antipsychotics

The notion that pharmacological treatment could favorably influence negative symptoms gained its foothold in the seminal work in the 1980s positioning clozapine as unique amongst antipsychotics in treatment-resistant schizophrenia (TRS). The so-called atypical antipsychotics that followed thereafter have been seen as sharing this same feature, including those more recently being investigated that highlight newer mechanisms of action [19]. Further to this last point, different theories have been posited to account for atypicality, including serotonin 5-HT_2_/dopamine D_2_ antagonism and fast dissociation from the D_2_ receptor; more recently, a third generation of antipsychotics has been designated, with aripiprazole the prototype, characterized by partial dopamine agonist properties [20]. While studies continue to compare newer antipsychotics, findings do not support a significant difference between atypical antipsychotics in the treatment of negative symptoms [21–23]. Evidence, to date, including several recent meta-analyses [15••, 21], aligns with other earlier studies indicating: (a) the newer antipsychotics are not superior to their conventional counterparts in the treatment of negative symptoms and (b) the effect in either case is modest.

### Antidepressants

Notwithstanding the challenges in distinguishing negative and depressive symptoms clinically, an extensive body of literature has accumulated on the efficacy of antidepressants in the treatment of negative symptoms. This has generated a number of meta-analyses addressing this topic, some of which look beyond antidepressants as a class to specific agents and/or groups categorized by mechanism of action. Two earlier meta-analyses provided equivocal evidence [24] and a lack of support [25], respectively, while the most recent report has supported some evidence of benefits that differs between agents [26]. A commentary on the collective work concluded that the evidence is not strong enough to support their use [27], although trials continue. For example, a recent 12-week placebo-controlled study evaluating buproprion, a dopamine and norepinephrine reuptake inhibitor as well as nicotine receptor antagonist, failed to identify clinical benefits [28]. In contrast, a 12-week RCT comparing reboxetine, a norepinephrine inhibitor (NRI), versus placebo reported a robust effect size in the reboxetine-treated group, although, notably, all subjects in this study were chronic and being treated with haloperidol as the antipsychotic [29]. A recently published meta-analysis looking only at mirtazapine, a noradrenergic and specific serotonergic antidepressant, concluded it demonstrates benefits in the treatment of negative symptoms, but trials included those where negative symptoms were not the primary outcome [30].

### CNS stimulants

Historically, such medications have been seen as contraindicated in individuals with psychosis because of risk of positive symptoms being induced or aggravated. Indeed, for a period of time, such drugs were employed as a challenge to establish risk of relapse [31]. The presence of concomitant antipsychotic treatment seems critical to this argument; for example, clinical data involving childhood schizophrenia and comorbid attention deficit disorder, where individuals received both antipsychotics and psychostimulants, indicated that such a combination was not associated with increased risk of psychosis [32]. The growing focus regarding negative symptoms and their treatment has renewed interest in this class of medications, and a recent review of the topic suggested evidence of benefits, adding that risk is reduced in clinically stable individuals with minimal positive symptoms and concomitant antipsychotic therapy [33]. While earlier trials employed drugs used routinely in attention deficit hyperactivity disorder (ADHD) (e.g., methylphenidate, *d*-amphetamine), there has been a shift more recently to trials with drugs used in the treatment of excessive sedation (modafinil, armodafinil). Of note, these drugs constituted a substantial contribution to the aforementioned review [33]; however, a recent meta-analysis reported differences with modafinil or armodafinil in the treatment of negative symptoms, but the effect size was small [34]. In addition, another RCT since noted no benefits with modafinil [35].

Recently, lisdexamfetamine (LDX), a pro-drug of amphetamine licensed for use in ADHD, has been investigated for its possible treatment of negative symptoms in schizophrenia. Favorable results were published based on an open-label, randomized withdrawal phase trial demonstrating improvement in negative symptoms with LDX and no indication of positive or negative symptom worsening with its abrupt discontinuation [36]. However, trials have since been terminated.

### Anticonvulsants

Reference has been made to these drugs in other reviews of negative symptoms [13, 18], and, like ECT, these drugs have a long history of augmentation use in schizophrenia as well as specific subpopulations (e.g., clozapine resistance, aggression) [37, 38]. In this context, improvement in negative symptoms as one of a number of outcome measures has been reported, but no controlled studies have specifically examined anticonvulsants for their utility in negative symptoms per se.

### Glutamate

This is one of several areas that has garnered considerable attention in recent years, not only in terms of negative symptoms but positive and cognitive symptoms as well [39•]. In terms of negative symptoms, numerous compounds, involving both ionotropic and metabotropic receptors, have been evaluated over the last two decades. Two meta-analyses, not specific to negative symptoms, suggested a favorable, albeit small, signal supporting the efficacy of drugs enhancing NMDA receptor function (e.g., *d*-serine, sarcosine, *N*-acetyl-cysteine, d-cycloserine), although the effect was different between agents [40, 41].

More recently, this line of thinking has spawned interest in other compounds that are presently being investigated, at least in part, for their benefits in negative symptoms. While trials continue, results, to date, have been mixed. Notably, a paper published in the last year offered ongoing support for *d*-serine [42], whereas several investigational compounds have been discontinued during early phase development, including agents that act through different mechanisms including glycine transporter 1 (GlyT1) inhibition (e.g., bitopertin) [43] and mGluR2/3-positive allosteric modulation (e.g., LY2140023) [44]. Further, there are agents designated as NMDA receptor antagonists that have also been the subject of investigation [45]. Once again, this speaks to the complexity of the glutamatergic system and differences between agents working through different mechanisms of action. Results with these strategies have as well been equivocal. Disappointing results with bitopertin and LY2140023 have led to their discontinuation for this indication [14, 46]. In the case of NMDA receptor antagonists, a meta-analysis examining amantadine and memantine did not support the utility of these medications in the treatment of negative symptoms, although the focus was not on negative symptoms specifically [45]. This said, an RCT published subsequently and looking at negative symptoms as the primary outcome reported that memantine led to significant improvement at 8 weeks, with a large effect size (Cohen’s d = 1.5 [95%CI 0.8–2.22]) [47].

Evidence continues to grow supporting a role for glutamate in processes critical to schizophrenia [48, 49], which may account for why, in the face of equivocal clinical evidence, efforts continue in developing new compounds. Suggestions to move the field ahead include the evaluation of other receptors, intracellular pathways, illness subtypes as well as stage of illness, and biomarkers/endophenotypes [39•, 50–54]. Again, though, this work is not confined only to efficacy regarding negative symptoms or, in fact, schizophrenia.

### Acetylcholine

As in the case of glutamatergic agents, the interest in this area is not specific to negative symptoms; indeed, much of this work has focused on cognitive symptoms as the primary endpoint. An earlier meta-analysis evaluating cholinesterase inhibitors in schizophrenia (rivastigmine, donepezil, galantamine) noted improvement in selected measures of cognition, but not negative symptoms [55]. A subsequent meta-analysis also had as its primary focus cognitive symptoms, and as an aside evaluated both glutamatergic and serotonergic receptors. It did note an effect on negative symptoms with two agents indicated in the treatment of Alzheimer’s disease, donepezil, and galantamine [56]; both share in common action as cholinesterase inhibitors while the latter is as well an allosteric modulator of nicotine acetylcholine receptors. A Cochrane review around the same time, and specific to cholinesterase inhibitors in schizophrenia, also suggested a signal for improvement in negative symptoms [57]; once more, though, negative symptoms were not the primary outcome. Further complicating interpretation of results is the need to disentangle the effect of changes in other domains on negative symptom scores [56], in addition to other pharmacological effects; for example, galantamine has been reported to enhance dopamine neurotransmission through its allosteric modulation of nicotinic acetylcholine receptors [58]. To date, no RCTs have been reported involving a cholinesterase inhibitor where negative symptoms were the primary outcome.

Efforts have also included a number of newer α7 nicotinic acetylcholine receptor (α7 nAChR) agonists/partial agonists as well as positive allosteric modulators [59–62]. To this point, though, it is difficult to evaluate the clinical benefits of this line of investigation. A recent double-blind trial involving galantamine/CDP-choline did not improve negative symptoms [63], nor did a recent phase 2 trial with the α7 nAChR agonist, TC-5619 [64]. Added to this is the early discontinuation of several other such agents [13]. In addition, there is again the issue of other mechanisms of action that may contribute to any effect seen; for example, preclinical evidence has shown that EVP-614, also a α7 nAChR agonist, increases cortical ACh and glutamate release, and this is true for dopamine, too [65]. As an aside, increased dopaminergic and noradrenergic activity has also been reported in association with CPD-choline administration [66].

## Serotonin

The notion that serotonin antagonism may prove useful in the treatment of negative symptoms gained momentum with the early claims that atypical antipsychotics, clozapine being the prototype, were superior to conventional antipsychotics in the treatment of negative symptoms [67]. It was postulated that their concomitant 5-HT_2_ antagonism played a role in this which, in turn, led to the development of selective 5-HT_2_ antagonists that were then investigated further in the treatment of negative (as well as positive) symptoms (e.g., ritanserin, M100907). While several RCTs involving ritanserin offered support for this line of investigation [68, 69], the more recent focus has turned to selective 5-HT_3_ antagonists (e.g., ondansetron, tropisetron, granisetron). Several trials specifically focused on negative symptoms have supported their efficacy [70, 71], as does a recent meta-analysis although it evaluated their effects on schizophrenia in general [72].

### Sex hormones

Gender differences have been well established in schizophrenia, with differences reported for age of onset, symptoms, course, and prognosis [73]. Once more, the work arising from this line of investigation is not specific to negative symptoms per se, but there is evidence specific to this topic, including a limited number of RCTs. As a starting point, various studies have identified relationships between neurosteroids/sex hormones and negative symptom severity [74–78]. In terms of RCTs, dehydroepiandrosterone (DHEA), an adrenal hormone involved in the production of androgens and estrogens, has been reported to improve negative symptoms [79], as have studies involving pregnenolone [80, 81]. Most recently, two further investigations specifically examining negative symptoms as part of their primary outcome have been added. In the first, a combination of pregnenolone and *l*-theanine over 8 weeks decreased both negative and anxiety symptoms, the co-primary outcome measures [82], while in the second the addition of raloxifene, an estrogen receptor modulator, decreased negative symptoms over 6 months in post-menopausal females with schizophrenia and prominent negative symptoms [83].

Currently, considerable attention is being given to oxytocin, and there are several recent reviews specific to schizophrenia [84, 85] in addition to a meta-analysis [86]. While much of the focus is on social cognition, its potential in the treatment of negative symptoms is highlighted in each. As of yet, there is a lack of studies where it has been specifically evaluated with negative symptoms as the primary focus.

## Inflammation-immunology

Kraepelin originally conceptualized schizophrenia as a neuroprogressive disorder (i.e., dementia praecox or “early dementia”) [87]. More recently, though, the focus has shifted to schizophrenia as a disorder of neurodevelopment, and within this framework, there is considerable interest in the role of inflammation and immune response [88]. Following this line of thinking, it is hypothesized that agents impacting the inflammatory response may prove useful in treating symptoms and, perhaps, altering the illness course. Only a limited amount of this work has been specific to negative symptoms, and in this regard, minocycline, a broad-spectrum tetracycline antibiotic with neuroprotective properties mediated through anti-inflammatory, anti-apoptotic, and antioxidant effects [89], has received the greatest attention. A recent meta-analysis supported its value in the treatment of negative symptoms, although this domain was not the primary outcome [90]. RCTs, where this is the case, have been mixed in their findings. There has been one negative trial [91], and another where only one domain, avolition (as measured using the SANS), was significantly impacted [92]. In contrast, two RCTs have reported positive results, one involving chronic schizophrenia [93] and one that looked at early-phase schizophrenia specifically [94]. Two large RCTs, the MINOS Trial and BeneMin, are currently underway, both focusing on early schizophrenia [95, 96]. Of note, there is interest in looking at schizophrenia across different symptom domains and stages of the illness (e.g., treatment resistance) [97], as well as other conditions where altering immune response may impact outcome (e.g., acute ischemic stroke) [98].

Compounds reviewed in other sections also have the capacity to act in a similar fashion. This would include neurosteroids such as pregnenolone (sex hormones), as well as compounds that modulate NMDA receptor-related inflammatory changes (e.g., *d*-serine, memantine) [42] (glutamate). Other drugs (e.g., ASA, Cox-2 inhibitors) would as well be seen as holding promise in this regard [18, 99]. Recent RCTs have been reported for dextromethorphan [100], methotrexate [101], interferon γ [102], atorvastatin as well as pravastatin [103], and pioglitazone [104] in schizophrenia, although none have focused specifically on negative symptoms. To date, one small RCT evaluating atorvastatin in negative symptoms has reported benefits [105].

### Brain stimulation

This is another line of investigation that currently generates considerable interest reflected in the number of meta-analyses [106–109] and reviews [110] specific to rTMS and negative symptoms in the last few years. Notably, the meta-analyses are consistent in the conclusion that rTMS is beneficial, with the effect size ranging from small and non-significant to as high as 0.80, the variability reflecting differences in moderators (e.g., study duration, stimulus frequency, outcome measure, illness duration) [106–109]. Further, a just published RCT indicated that response with active treatment is sustained over a 24-week follow-up [111]. However, another recently published RCT (*n* = 175) failed to establish a beneficial effect with active versus sham rTMS [112••]. Given the many moderators that can impact outcome, it is difficult to reconcile these findings with the numerous earlier reports and meta-analyses, but the most recent investigation is by far the largest in terms of sample size.

Going forward, there is interest in deep rTMS and its impact on such features as negative symptoms in schizophrenia [113], but it remains for future investigations to address this line of investigation. Transdirect current stimulation (tDCS) is being pursued in the treatment of schizophrenia, and a recent review has concluded that it may prove useful for negative symptoms [114]; to date, however, there is only one small RCT published, although results are favorable regarding negative symptoms [115].

Other forms of brain stimulation remain of interest, but as of yet have been the subject of much less investigation. Electroconvulsive therapy (ECT) has a long history as a treatment in schizophrenia, although at this point is largely confined to augmentation in TRS; in fact, it is amongst the recommended options in the face of partial clozapine response (i.e., ultra-resistant schizophrenia) [116]. While TRS is generally characterized by positive symptoms, a small body of literature has recently reported success in the management of TRS with prominent negative symptoms [117–119]. However, these preliminary results have not been corroborated by RCTs.

Repeated vagus nerve stimulation (rVNS) has been identified as useful in other medical and psychiatric conditions (e.g., epilepsy, depression), and a recent randomized, sham controlled and double-blind study evaluated its efficacy in schizophrenia, although efficacy was not established across any outcome measure, including negative symptoms [120]. Deep brain stimulation (DBS) has also found a role in the treatment of different medical and psychiatric conditions, and while there is interest and a rationale for use in negative symptoms [121], no such studies have been published as of yet.

## Conclusions

Table 1 summarizes the literature discussed. As concluded in the recently published meta-analysis [15••], there is insufficient evidence at present to support a specific treatment for negative symptoms. This is despite a tremendous increase in interest in the topic, as well as studies where negative symptoms are the identified primary outcome.

**Table 1:** Treatment of negative symptoms

Topic	Literature	Comments
Comprehensive meta-analysis	A meta-analysis of RCT interventions to December 2013 involving 168 trials (*N* = 6503 in treatment arm, *N* = 5815 in placebo arm).	Treatments evaluated included antipsychotics (AP, first and second generation), antidepressants (AD), pharmacological combinations (e.g., AP + AP; AP + AD), glutamatergic agents, brain stimulation, and psychological interventions. Some differences were statistically significant but none reached threshold for clinical significance [15••]
Specific somatic interventions		
Antipsychotics	The introduction of “atypical” antipsychotics came with claims of superior efficacy in the treatment of negative symptoms. This had been identified with clozapine in trials evaluating its efficacy in treatment-resistant schizophrenia	Modest improvement, not clinically significant, may be observed with antipsychotic treatment, possibly related to efficacy on other symptom domains and/or dopamine “sparing”. Two recent meta-analyses, one specific to negative symptoms, do not support superiority of the newer antipsychotics [15••, 21]. Going forward, the notion of developing an agent that can significantly and simultaneously impact the different symptom domains of schizophrenia seems unlikely
Antidepressants	This work has been built around augmentation with SSRIs and, more recently, the newer classes of antidepressants that have followed	Only one of three earlier meta-analyses focused on negative symptoms offered support for such an approach [24–26]. The fourth and most recent meta-analysis also did not identify changes that were clinically significant [15••]. This said, ADs are used routinely with APs in schizophrenia and as they evolve it is likely that new ADs will be evaluated in negative symptoms
CNS stimulants	This literature has included trials involving ADHD drugs and, more recently, drugs indicated in the treatment of excessive sedation (e.g., modafanil). Lisdexamfetamine, indicated for ADHD, was recently evaluated for a possible indication in negative symptoms but this line of investigation has been terminated	Collectively, this line of investigation has established that such agents can be used safely in individuals with psychosis. A review in 2013 specific to negative symptoms concluded evidence supports larger trials be done [33], although a more recent meta-analysis confined to modafanil/armodafanil reported only a small effect size [34]
Anticonvulsants	Anticonvulsants are frequently used in schizophrenia and it is in this context that a potential effect on negative symptoms has been reported	This area has not generated a lot of interest. To date, there are no published RCTs specifically evaluating this class of medications in trials focused on negative symptoms
Glutamate	This line of investigation has garnered a great deal of research although drawing conclusions is complicated by different mechanisms of action. Much of this work is not confined to negative symptoms per se	Two earlier meta-analyses, not specific to negative symptoms, suggested these drugs could be effective [40, 41], and a recent small RCT (*N* = 35) involving d-serine supported this position [42]. However, trials with agents working through other mechanisms (e.g., bitopertin, LY2140023) have been terminated for this indication. A meta-analysis examining NMDA antagonists (8 studies, *N* = 406) did not support their use in negative symptoms [45], but a small RCT (*N* = 40) since reported a large effect size [47]. The recently published meta-analysis looking at numerous treatments did not report clinically significant results for glutamatergic agents as a class [15••]. Despite these mixed results, work along these lines is likely to continue
Acetylcholine	This focus has also garnered considerable interest in recent years, but once again the focus is not specific to negative symptoms. The research can be divided into work involving cholinesterase inhibitors (e.g., donepezil) and a newer group of α7 nAChR agonists/partial agonists as well as positive allosteric modulators	Three meta-analyses examining the cholinesterase inhibitors, but not specific to negative symptoms, have suggested potential benefits [55–57] but no such RCTs have been published as of yet. Taken together, results with the newer α7 nAChR agents have not been favorable, and it is presently unclear if efforts specific to negative symptoms will be continued
Serotonin	Earlier work involving selective 5-HT_2_ antagonists (e.g., ritanserin) has given way to research focused on 5-HT_3_ agents (e.g., ondansetron)	A recent meta-analysis looking at different domains in schizophrenia has offered support for this approach [72], as have several RCTs specific to negative symptoms [70, 71]
Sex Hormones	Support for this line of investigation arises from work identifying a relationship between neurosteroids/sex hormones and negative symptom severity, in addition to treatment trials	A small number of trials specific to negative symptoms, and utilizing different agents (DHEA, pregnenolone, raloxifene) have been carried out, with favorable results [80–83]. Oxytocin is receiving considerable attention at present, particularly in terms of measures of social cognition. A recent meta-analysis indicated it may have a role to play in schizophrenia and alluded to some evidence regarding its value in negative symptoms [86]. As of yet, though, specific trials of this sort have not been published
Inflammation/Immunology	This particular research aligns closely with the shift in focus to neurodevelopmental models of schizophrenia and, like other areas discussed, is not specific to negative symptoms or, in fact, schizophrenia	Much of the work to date has involved minocycline, with RCTs specific to negative symptoms providing mixed results [91–94]. Compounds reviewed under other categories here (e.g., *d*-serine [Glutamate); pregnenolone [Sex Hormones]) may, at least in part, establish their response through these mechanisms as well. Larger trials with minocycline are underway [95, 96], and it can be expected that other agents will be evaluated in trials where negative symptoms are the primary outcome
Brain Stimulation	Brain stimulation, including both ECT and rTMS, has a history in refractory forms of schizophrenia (e.g., TRS, refractory hallucinations) although considerable work with rTMS is now focused on negative symptoms	While earlier meta-analyses supported the efficacy of rTMS in negative symptoms [106–109], the largest trial to date, just published, failed to find benefits [112••]. A number of identified moderators of response complicate comparison across studies, and newer techniques (e.g., dTCS, DBS) have little or no data. Given the evidence to date, conflicting as it is, further studies of this sort are likely to continue

*AD* antidepressant, *ADHD* attention deficit hyperactivity disorder, *AP* antipsychotic, *DBS* deep brain stimulation, *dTCS* direct transcranial stimulation*, 5HT* serotonin, *nAChR* nicotinic acetylcholine receptor, *NMDA N*-methyl-d-aspartate, *RCT* randomized controlled trial, *rTMS* repetitive transcranial magnetic stimulation, *SSRI* selective serotonin reuptake inhibitor

A number of factors may contribute to the lack of success to date. Diagnostically, it is difficult distinguishing primary from secondary negative symptoms, just as it is a challenge differentiating negative symptoms from other psychiatric diagnoses such as depression. Our understanding regarding underlying pathophysiological processes is not well established, resulting in strategies for treatment that are, at best, speculative. This is perhaps best captured by the numerous trials that choose to assess multiple symptom domains rather than focusing specifically on negative symptoms. From a conceptual standpoint, the very definition of negative symptoms represents a work in progress, and it has been demonstrated that outcomes can be influenced by the measures employed [16, 122]. More recently, guidelines have been forwarded regarding study trial design [123•], but, in fact, much of the work to this point falls short of these standards. It is now common to isolate different components under the framework of negative symptoms [6], but this strategy is in its earliest stages and it is unclear how these may differentially respond to treatment. We continue to seek biomarkers and/or endophenotypes that may not only improve diagnosis but, in addition, advance the field in clinical subtyping; already, different trajectories have been reported [124] but this work too is in its earliest stages. It has been established that negative symptoms predate the onset of positive symptoms, and there is speculation that aberrations occurring during neurodevelopment are responsible. This underscores the importance of timing of interventions, and expectations regarding the success of treatments that are implemented later in the illness course. Indeed, it is possible that effective treatments await that time when we can reliably identify those who will go on to develop schizophrenia, which would also permit interventions well in advance of when diagnosis occurs, that is when psychotic features are evident for the first time. Where benefits have been recorded to this point, the reported effect size is often not of a magnitude to be clinically significant [15••]. Finally, there is evidence that non-biological factors may play a role [125, 126], which raises questions as to the limitations of somatic interventions and the need to investigate their benefits in combination with non-biological strategies.

Future drug development in the field of schizophrenia clearly identifies negative symptoms as an important unmet need [127]. This is undoubtedly driven, at least in part, by evidence that negative symptoms play a critical role in the functional decline observed in many individuals with schizophrenia, a decline that is not necessarily addressed with adequate control of positive symptoms. What is less clear, however, is what lines of investigation hold promise of success or, in fact, whether effective strategies can be developed until we are able to reliably diagnose schizophrenia and implement treatments earlier in the illness’ evolution.
